# Effectiveness of promotion and support for physical activity maintenance post total hip arthroplasty—study protocol for a pragmatic, assessor-blinded, randomized controlled trial (the PANORAMA trial)

**DOI:** 10.1186/s13063-022-06610-4

**Published:** 2022-08-13

**Authors:** Theresa Bieler, S. Peter Magnusson, Volkert Siersma, Mie Rinaldo, Morten Torrild Schmiegelow, Torben Beck, Anne-Mette Krifa, Birgitte Hougs Kjær, Henrik Palm, Julie Midtgaard

**Affiliations:** 1 Department of Physical and Occupational Therapy, Copenhagen University Hospital - Bispebjerg and Frederiksberg, Bispebjerg Bakke 23, Nielsine Nielsens Vej 10, Building 10, 2400 Copenhagen, NV Denmark; 2 Institute of Sports Medicine Copenhagen, Department of Orthopedic Surgery, Copenhagen University Hospital – Bispebjerg and Frederiksberg, Bispebjerg Bakke 23, Nielsine Nielsens Vej 8, Building 8, 2400 Copenhagen, NV Denmark; 3 The Research Unit for General Practice and Section of General Practice, Department of Public Health, University of Copenhagen, Øster Farimagsgade 5, building 24, entrance R, 1353 Copenhagen K, Denmark; 4 Department of Orthopedic Surgery, Copenhagen University Hospital - Bispebjerg and Frederiksberg, Bispebjerg Bakke 23, Nielsine Nielsens Vej 6, Building 6, 2400 Copenhagen, NV Denmark; 5 Gartnervænget 1, 3100 Hornbæk, Denmark; 6 Department of Clinical Medicine, University of Copenhagen, Blegdamsvej 3B, 2200 Copenhagen N, Denmark; 7 Centre for Applied Research in Mental Health Care (CARMEN), Mental Health Centre Glostrup, Nordstjernevej 41, 2600 Glostrup, Denmark

**Keywords:** Total hip arthroplasty, Behavior change intervention, Motivation, Pedometer, Physical activity, Physical function, Randomized controlled trial

## Abstract

**Background:**

Total hip arthroplasty is considered an efficacious procedure for relieving pain and disability, but despite that objectively measured physical activity level remains unchanged compared to pre-surgery and is still considerably lower than that of a healthy age- and sex-matched population 6–12 months post-surgery. Since there is a graded relationship between physical activity level and functional performance, increasing physical activity may enhance the outcome of the procedure. This study aims to investigate whether promotion and support of physical activity initiated 3 months after total hip arthroplasty complementary to usual rehabilitation care can increase objective measured physical activity 6 months post-surgery.

**Methods:**

The trial is designed as a pragmatic, parallel group, two-arm, assessor-blinded, superiority, randomized (1:1), controlled trial with post intervention follow-up 6 and 12 months after total hip arthroplasty. Home-dwelling, independent, and self-reliant patients with hip osteoarthritis are provisionally enrolled prior to surgery and re-screened about 2–3 months post-surgery to confirm eligibility. Baseline assessment is conducted 3 months post-surgery. Subsequently, patients (*n*=200) are randomized to either a 3-month, multimodal physical activity promotion/education intervention or control (no further attention). The intervention consists of face-to-face and telephone counselling, patient education material, pedometer, and step-counting journal. The primary outcome is objectively measured physical activity, specifically the proportion of patients that complete on average ≥8000 steps per day 6 months post-surgery. Secondary outcomes include core outcomes (i.e., physical function, pain, and patient global assessment) and health-related quality of life. Furthermore, we will explore the effect of the intervention on self-efficacy and outcome expectations (i.e., tertiary outcomes).

**Discussion:**

By investigating the effectiveness of a pedometer-driven, face-to-face, and telephone-assisted counselling, behavior change intervention in complementary to usual rehabilitation, we hope to deliver applicable and generalizable knowledge to support physical activity after total hip arthroplasty and potentially enhance the outcome of the procedure.

**Trial registration:**

www.clinicaltrials.govNCT04471532. Registered on July 15, 2020.

## Background

Osteoarthritis (OA) is a painful, chronic joint disease, that often involves several joints. Hip OA is one of the leading causes of global disability [1], and the odds of frailty are four times higher among older adults with hip OA than those without OA [2]. The prevalence of hip OA increases consistently with age [1], and for patients with advanced disease or substantial symptoms that do not respond to other treatments, total hip arthroplasty (THA) is the choice of treatment [3].

Total hip arthroplasty is considered an efficacious procedure for relieving pain and disability, but despite that objectively measured physical activity level remains unchanged compared to pre-surgery and is still considerably lower than that of a healthy age- and sex-matched population 6–12 months post-surgery [4, 5]. Six to eight months after THA, physical function is only recovered to about 80% of that of healthy peers [6], and older adults (≥ 65 years) still seem to be at increased risk of frailty [7]. This lack of completely regained functional status could possibly be related to the higher healthcare costs that these patients continue to impose despite their THA [8]. Since there is a graded relationship between physical activity level and functional performance [9], increasing physical activity after THA may enhance the outcome of the THA. This could also be a simple and relatively inexpensive method for improving general health [10] as patients with OA can have extensive comorbidity, e.g., joint pain, hypertension, cardiovascular diseases, obesity, respiratory diseases, and diabetes [11, 12], which all are diseases where physical activity has a positive impact.

Lack of increase in physical activity despite increased capability after THA may be related to the sedentary behavior adopted by the patients prior to surgery [5], experiences of pain in other joints or other limitations related to comorbidities, or uncertainty [13, 14]. According to a systematic review, patient-reported barriers to engaging in physical activity after THA are largely related to limited or inadequate information or education resulting in uncertainty about “doing the right thing” for both the individual’s recovery and the longevity of the joint replacement [13].

While patients’ adherence to exercise therapy tends to drop over time [15, 16], promotion of increase in physical activity is not explicitly addressed in current post-surgical physiotherapy practice. Few studies [17–21] have investigated the effect of adding specific interventions to increase physical activity to usual rehabilitation care after THA or total knee arthroplasty (TKA). Specifically, two feasibility studies [17, 18] and a larger randomized controlled study (RCT) [21] have shown positive effects on physical activity from a 6-month [17], 12-week [18], or 6-week [21] intervention, including self-monitoring of physical activity behavior and receiving real-time feedback from physical activity trackers in combinations with daily [18, 21] and or weekly step goals [17, 18, 21], action planning and monthly group support meetings [18], and monthly follow-up phone calls [17]. Moreover, a large RCT [20] showed significant and clinically relevant improvement in physical activity from a 6-month intervention that included telephone health coaching (weekly/biweekly) and financial incentives. In contrast, a smaller pseudo-randomized study [19] showed no additional effect of encouraging patients to increase their daily step count by 5% twice weekly for 3 weeks (no continuous feedback).

The behavioral change wheel [22] is as a relatively new method for characterizing and designing behavioral change interventions (a coordinated set of activities designed to change specified behavior change patterns) where capability, opportunity, and motivation interact to generate behavior. Capability is defined as the individual’s psychological and physical capacity to engage in the activity concerned, e.g., having the necessary knowledge and skills [22]. Opportunity is defined as all the extra-personal factors that make the behavior possible or prompt it. Motivation is defined as all those brain processes that energize and direct behavior, not just goals and conscious decision-making [22]. Furthermore, these three behavioral sources are linked to nine “intervention functions” aimed at addressing deficits in the behavioral sources [22].

The timing of a behavior change intervention may influence the rate of physical activity gain [18], but the optimal time point for a behavior change intervention after THA is unclear [19]. The inpatient rehabilitation period has been suggested as the earliest time point for a cost and time effective physical activity intervention, because at that time point the patients are more condensed and approachable compared to immediately after surgery [19]. The outpatient rehabilitation period has also been proposed to offer a unique opportunity to change patients’ attitudes and regular behaviors regarding physical activity [20], but at that time point, the patient’s physical capacities and focus could be fully claimed by the rehabilitation program [19].

In the present RCT, the PANORAMA (promotion and support for physical activity maintenance post total hip arthroplasty) trial, we investigate the effectiveness of adding a pragmatic, pedometer-driven physical activity promotion intervention to usual rehabilitation care 3 months after THA. The intervention addresses the patients’ uncertainty regarding physical activity and build upon the promising results using physical activity sensor feedback in combination with other techniques such as goal setting and telephone health coaching.

## Methods/design

### Primary objective

The primary objective of this RCT is to investigate the effectiveness of a physical activity behavior change intervention, initiated 3 months after THA complementary to usual rehabilitation care compared to control (i.e., usual rehabilitation care with no further attention), on objectively measured physical activity 6 months after surgery in patients who had a THA because of hip OA.

### Secondary and other prespecified objectives

**Fig. 1:**
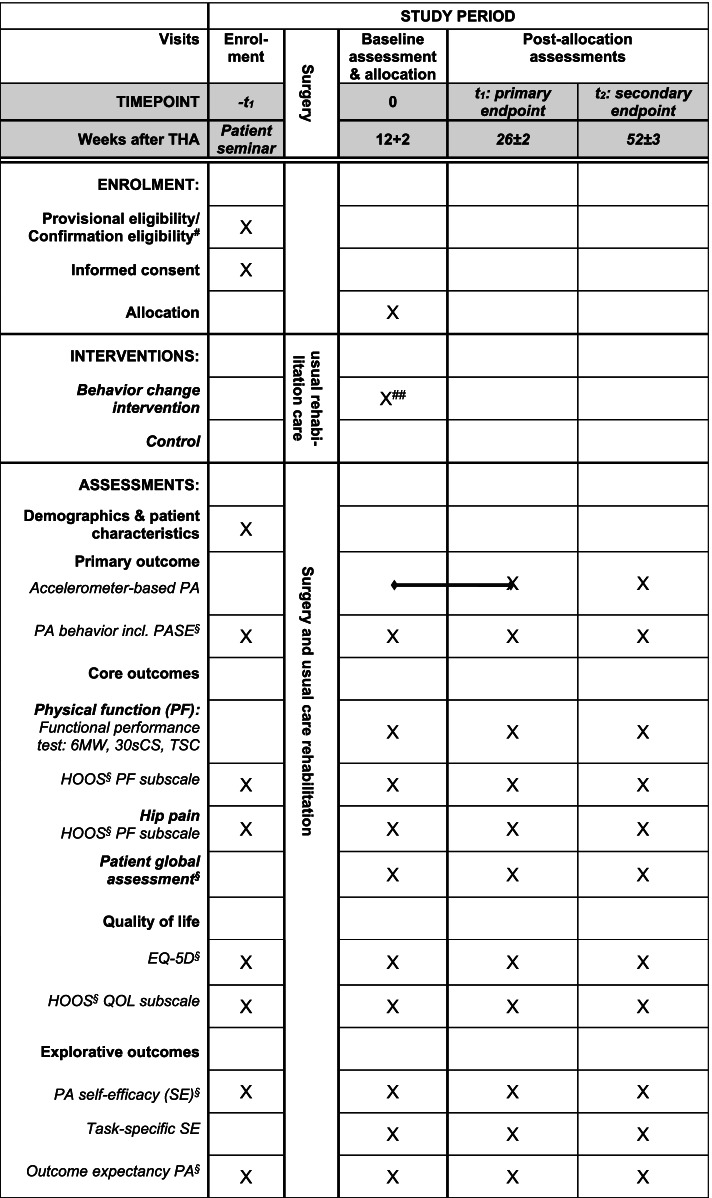
SPIRIT diagram for trial stages of enrolment, intervention, and assessment. ^#^Telephone-based 2 months + 2 weeks after surgery; ^##^two telephone-assisted counselling 3 weeks±1 respectively 4 weeks±1 after the initial face-to-face counselling; ^§^questionnaire. Abbreviation: PA: physical activity; PASE: the Physical Activity Scale for the Elderly; the 6MW: 6-min walk test; the 30sCS: 30-s chair stand test; TSC: timed stair climbing; HOOS: the Hip disability and Osteoarthritis Outcome Score; QOL: Quality Of Life; OEE-2: the Outcome Expectancy for Exercise scale-2

**Fig. 2:**
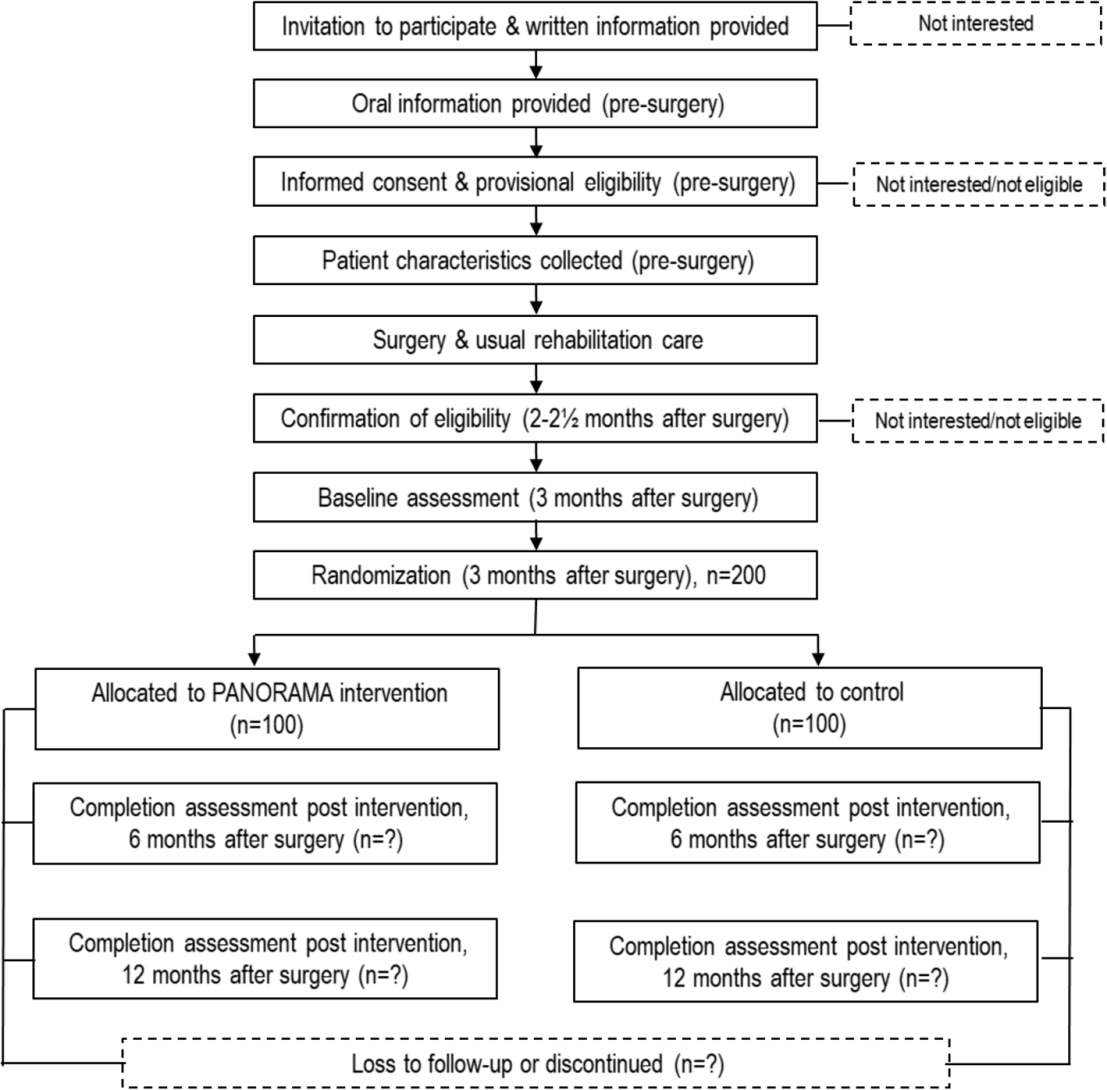
Flow of participants

The secondary key objectives are to investigate the effects of the intervention on core outcomes (i.e., physical function, hip pain, and global perceived effect [23, 24]), health-related quality of life, and exploratory outcomes (i.e., outcome expectancy for physical activity, self-efficacy for physical activity, and task-specific self-efficacy) 6 months after THA (Figs. 1 and 2). Other prespecified objectives are to investigate the long-term effects on the abovementioned outcomes 12 months after THA (Figs. 1 and 2). In addition, we will conduct a qualitative sub-study embedded in the RCT to further understanding of the potential change mechanisms of the PANORAMA intervention.

### Hypothesis

We hypothesize that the intervention will increase the proportion of participants that complete on average ≥8000 steps per day 6 months post-surgery to 50% compared to 30% after usual rehabilitation care.

### Design

**Fig. 3:**
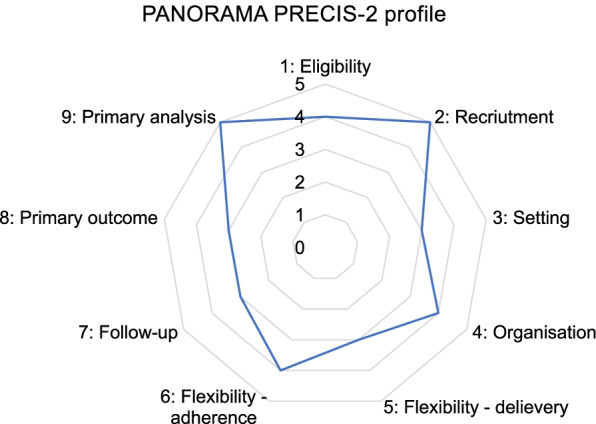
The PRagmatic-Explanatory Continuum Indicator Summary – PRECIS-2 wheel for the trial domains. 1: Very explanatory; 2: Rather explanatory; 3: Equally pragmatic/explanatory; 4: Rather pragmatic; 5: Very pragmatic

The PANORAMA trial is designed as a pragmatic (Fig. 3), parallel group, two-arm, assessor-blinded, superiority, randomized (1:1), controlled trial with post intervention follow-up 6 and 12 months after THA (Figs. 1 and 2). The patients are provisionally enrolled in the study prior to surgery and re-screened about 2–3 months after surgery to confirm eligibility. Baseline assessment is conducted 3 months after THA and subsequently the patients are randomized (Figs. 1 and 2). The trial protocol is conducted according to the guidance for protocols of clinical trial, Standard Protocol Items: Recommendations for Interventional Trials (The SPIRIT Statement) [25] and the description of the intervention follow the Template for Intervention Description and Replication (TIDieR) [26].

### Study setting

The study takes place at Bispebjerg and Frederiksberg Hospital (BFH), the orthopedic department and outpatient clinic, and the department of physical and occupational therapy.

### Participants

#### Eligibility criteria

Patients scheduled for THA at BFH are consecutively recruited from the end of August 2020 with anticipated completion of recruitment in December 2022. A patient is eligible for study participation if he/she meets the following criteria: (1) home-dwelling, independent, and self-reliant adult, (2) undergone primary THA because of hip OA, (3) has signed informed consent to participate. A participant is excluded from the study if he/she meets any of the following criteria: (1) planned joint arthroplasty in the lower extremities within the next 6 months, (2) are unable to read, understand, and speak Danish, (3) complications in relation to THA, e.g., dislocation, fracture, or infection, (4) any other condition that in the opinion of the investigator makes a potential participant unfit for participation.

#### Recruitment, consent, screening, and enrolment

Patients are recruited through the orthopedic outpatient clinic and the patient seminars at orthopedic department M50. The patients receive verbal and written information by the surgeons at the preoperative consultation at the orthopedic outpatient clinic. Prior to surgery at the patient seminars at the orthopedic department M50, the patients are invited to participate in the study by the principal investigator (TB), or in her absence by a designated delegate (MR). The patients receive a full explanation of the study design and study procedures, and interested patients provide written informed consent and undergo the pre-screening procedures. Afterwards TB or MR collects patient characteristics and hands out the pre-surgery questionnaires (further described in the outcome and assessment section and Fig. 1). However, due to Corona pandemic, the patient seminars have been closed and instead the patients receive the information about the time before, during, and after the THA through videos at the hospital’s home page and a telephone call from the staff at orthopedic department M50. Consequently, we adjusted the recruitment procedure so that verbal information, written informed consent, the pre-screening procedures, and provisionally enrollment in the study are conducted prior to surgery in connection with Corona testing at BFH 2 days before surgery or at the day of surgery at orthopedic department M50. Approximately 2 months after surgery, where it is expected that most of the participants have received and completed the conventional outpatient physiotherapy as per usual care, the participants are contacted by phone and screened again by TB to confirm eligibility (Fig. 1).

### Intervention

The patients are randomized to the PANORAMA intervention or control (no further attention).

#### The intervention group: the PANORAMA intervention package

The intervention is a 3-month package (Table 1) initiated 3 months after THA including (1) a brief motivational interviewing regarding physical activity, (2) patient educational material regarding physical activity after THA (a video and a leaflet), (3) a pedometer (Garmin, Vivofit 4), (4) a step-counting journal, (5) a practice-oriented leaflet regarding how to use the pedometer, the step-counting journal, and goal setting as well as strategies to incorporate physical activity into daily life, and (6) telephone-assisted counselling (for more details see Tables 1 and 2).

**Table 1:** Overview of the PANORAMA intervention

Consultations	Content
One initial, face-to-face, physical activity counselling. Duration: 45 min. Time point: immediately after baseline assessment and randomization 3 months after THA.	• A brief motivational interviewing regarding physical activity (10 min) • Patient education regarding physical activity after total hip arthroplasty including recommendations and safety. This session is based on an “orthopedic surgeon guided” video (5 min) and a leaflet (in total 10 min) • Handling out pedometer and educational material. Based on a practice-oriented leaflet practical advice on how to use a pedometer, a step-counting journal and goal setting as well as strategies to incorporate physical activity into daily life (in total 20 min) • Goals (5 min)
Two telephone-assisted counselling Duration: 20 min each. Time point: after 3 respectively 7 weeks.	• Experiences with physical activity since last counselling? • How are the tools working? • Renewed goals

**Table 2:** Detailed description of the PANORAMA intervention package’s components

Component	Description
The brief motivation interviewing	Includes discussion of (1) personal experience with PA after THA, (2) motivation and barriers related to PA adaptation and adherence, and (3) the patient education material regarding PA after THA (video and leaflet).
Patient educational material regarding PA after THA	A 6-min surgeon-led patient education video, which summarize the content of a leaflet signed by the patients surgeon (picture on the front page) regarding PA after THA and PA recommendations, which mentions all the benefits of being physically active on recovery of physical function and, e.g., health including recommendations of the right things to do both for individual’s recovery and the longevity of the joint replacement [27, 28]. To provide examples, experiences and empower the patients to succeed engaging in PA patients (*n*=3) like themselves participate in the video.
Information leaflet about how to use the pedometer, step-counting journal and goal setting and strategies to increase daily PA	Leaflet-based information and demonstration of (1) how to wear and use a pedometer, (2) how to use a step-counting journal, and (3) how to set goals and make action planning. The participants are instructed to use the first week as an observation period to give them insight into how many steps their current daily life practice translates to. After the first week, the patients are encouraged to determine daily step goals on a weekly basis. In addition, the patients are encouraged to create detailed plans on in which situation and/or where to act to increase their physical activity level [29] and be SMART (Specific, Measurable, Achievable, Relevant and Timed) with their goals, (4) tips on how to increase PA and daily steps and reduce sedentary time by making small changes and other strategies to increase daily PA and links to other PA resources. Participants from minimal contact pedometer-based interventions to promote walking have emphasized that future interventions should provide more examples and strategies for meeting PA recommendations or more strategies for addressing barriers to PA [30, 31].
Pedometer	The participants receive a pedometer (Garmin, Vivofit 4) and are encouraged to use the pedometer to monitor the number of steps walked each day during all waking hours.
Step-counting journal	The participants receive a printed step-counting journal (alternatively an electronic version) and are instructed to record date and total number of steps displayed on the pedometer in their journal at the end of each day [29]. In addition, to calculate and fill in additional steps for activities such as bicycling and other activities that are not well recorded by the pedometer. Furthermore, on a weekly basis to calculate and register the mean number of daily steps in the journal. Finally, to register the goal for the following week.
Telephone-assisted counselling (*n*=2)	The two telephone-assisted counselling for PA are follow-ups on the initial face-to-face counselling. The content is now based on the participants’ new experience with PA during the first weeks respectively the first 7 weeks and how the tools are working and strategies for continued PA participation.

Abbreviations: *THA* total hip arthroplasty, *PA* physical activity

Three, trained, physiotherapists from the department of physical and occupational therapy deliver the PANORAMA intervention, i.e., one 45-min face-to-face physical activity counselling immediately after randomization (Fig. 1, Table 1) followed by two 20-min telephone-assisted counselling, respectively 3 weeks (± 1 week) and 7 weeks (± 1 week) later (Table 1). Detailed protocols for all counselling have been developed.

#### Theoretical framework

The behavioral change wheel [22] was used to design the intervention. After selecting the intervention functions (education, persuasion, enablement, training, modelling) most likely to be effective in changing physical activity behavior in patients after THA (Table 3), we have linked these to more fine-grained specific behavior change techniques (BCTs) [32] (Table 4).

**Table 3:** Links between the PANORAMA intervention components and behavior sources and intervention function

Components of the intervention	Behavior sources	Intervention function
PA counselling/motivational interviewing (including self-selected physical activities and incremental goal setting)	Motivation: reflective and automatic Capability: psychological and physical	Education, persuasion, enablement, training
Surgeon-led patient education video regarding PA after THA	Motivation: reflective and automatic Capability: psychological	Education, persuasion, enablement, modelling
Leaflet (signed by surgeons (+picture)) regarding PA after THA (OA) including recommendations/the right things to do both for individual’s recovery and the longevity of the joint replacement	Motivation: reflective and automatic Capability: psychological	Education, persuasion, enablement
Information leaflet about how to use the pedometer, step-counting journal, and goal setting and strategies to increase daily PA	Motivation: reflective and automatic Capability: psychological	Education, persuasion, training, enablement
Pedometers	Motivation: reflective Capability: psychological	Enablement
Step-counting journal	Motivation: reflective Capability: psychological	Enablement, education, training
Telephone-assisted counselling	Motivation: reflective and automatic Capability: psychological and physical	Education, persuasion, enablement, training

Definitions: Education= Increasing knowledge; Persuasion= Using communication to induce positive or negative feelings or stimulate action; Training= Imparting skills; Enablement= Increasing means/reducing barriers to increase capacity or opportunity; Modelling= Providing an example for people to aspire to or imitate

Abbreviations: *PA* physical activity, *THA* total hip arthroplasty, *OA* osteoarthritis

**Table 4:** Specification of the potent behavior change techniques (BCTs) used in the PANORAMA intervention

Components of the intervention	Behavior change techniques
PA counselling/motivational interviewing	1.1 Goal setting (behavior) 1.2 Problem solving 1.4 Action planning 8.7 Graded task 9.2 Pros and cons 15.1 Verbal persuasion about capabilities
Surgeon-led patient education video regarding PA after THA	4.1 Instruction on how to perform the behavior 5.1 Information about health consequences 5.6 Information about emotional consequences 6.1 Demonstration of the behavior 6.3 Information about others approval 8.2 Behavior substitution 8.7 Graded tasks 9.1 Credible source 15.1 Verbal persuasion about capabilities
Leaflet (signed by surgeons (+picture)) regarding PA after THA	4.1 Instruction on how to perform the behavior 5.1 Information about health consequences 5.6 Information about emotional consequences 8.2 Behavior substitution 9.1 Credible source
Information leaflet about pedometer, step-counting journal, goal setting, and strategies to increase daily PA	4.1 Instruction on how to perform the behavior 5.1 Information about health consequences 6.1 Demonstration of the behavior 6.3 Information about others approval 8.2 Behavior substitution 8.3 Habit formation 8.4 Habit reversal 8.7 Gradual tasks 13.2 Framing/reframing
Pedometers	2.4. Self-monitoring of outcome of behavior 7.1 Prompts/cues
Step-counting journal	2.3 Self-monitoring of behavior 2.4. Self-monitoring of outcome of behavior 7.1 Prompts/cues
Telephone-assisted counselling	1.1 Goal setting (behavior) 1.2 Problem solving 1.4 Action planning 1.5. Review behavior goals 2.7 Feedback on outcome(s) of behavior 8.7 Graded task 9.2 Pros and cons 10.4 Social reward 15.1 Verbal persuasion about capabilities

Abbreviations: *PA* physical activity, *THA* total hip arthroplasty

#### Engagement of patients

Three patient partners (including AK) have participated in the development of the PANORAMA trial and particularly in the development of the intervention. To further ensure the relevance and feasibility of the intervention, we performed a prospective, qualitative, pilot study based on a telephone interview with patients (including the patient partners) before (*n*=10) and 2 months after (*n*=7) THA. To further incorporate the users’ perspective, three focus group interviews were conducted: two (*n*=3, the patient partners) during development of the educational material, and one to evaluate the content, duration, and timing of the intervention with patients (*n*=3) who had pilot-tested the intervention 3 months after their THA.

#### Usual care

In Denmark, THA surgery is fast-track surgery [33]. At BFH, the posterior approach is used, which is the most used approach in Denmark. Average length of stay is 1–2 days, and discharge is predominantly directly to the patient’s home. Early postoperative mobilization is encouraged, and in general, the patients have no movement- or weight-bearing restrictions. On the first postoperative day, a physiotherapist provides the patient with walking aid (typically crutches) and information and guidance regarding mobilization to achieve early functional independence postoperatively. In addition, the patient receives instruction in a home-based exercise program addressing impairments in hip range of motion and lower extremity muscle function. Standard care includes prescription of outpatient physiotherapy. According to the Danish Health Authority’s national clinical practice guideline [34], in general, the patients are offered one or two outpatient sessions of exercise instructions after THA, because supervised exercise is not significantly more effective than non-supervised home-based exercise for improving physical function, pain, and health-related quality of life [34, 35]. However, there is a great variation in content and duration of the outpatient physiotherapy offered to patients [34].

#### Criteria for discontinuing or modifying allocated interventions

Standard criterion for intervention discontinuation is withdrawal of participant consent. Otherwise, there will be no special criteria for discontinuing or modifying allocated interventions.

### Outcomes and assessment

Data are collected on four occasions at the BFH: once prior to THA (some days before or at the day of surgery) and three times after THA ((3 months + 2 weeks (baseline), 6 months ± 2 weeks (post intervention), and 12 months ± 3 weeks after surgery (follow-up)) (Figs. 1 and 2).

### Primary outcome measure

The primary outcome is objectively measured physical activity determined as the proportion of participants that on average complete ≥ 8000 steps per day 6 months after THA measured by the SENS motion® system [36]. To further understand how the PANORAMA intervention affects physical activity and sedentary behavior (physical inactivity), participants are asked to wear the accelerometer continuously during the entire intervention period (3 to 6 months after THA) (Fig. 1). Only the mean number of steps per day during the last week (7 days) will be used for data analysis of the primary outcome. The latter is unknown to the participants. At follow-up, participants are asked to wear the accelerometer for 1 week after the 12-month follow-up visit (Fig. 1).

The SENS motion system consists of a waterproof sensor (50×21×5 mm, weight 8 g) with a triaxial accelerometer, sampling acceleration at 12.5Hz, with a range of ±4𝐺, embedded within a small Band-Aid (Medipore^TM^, 3M, Soft Cloth Surgical Tape on Liner) to be worn discretely on the lateral aspect of the thigh (approximately 10 cm from the lateral epicondyle of the knee), connected wirelessly (Bluetooth technology) to a dedicated smartphone or tablet application for data uploading [36]. This system can measure movements or free-living physical activity continuously 24 h a day. The sensor has a battery lifespan of approximately 20 weeks and a built-in memory capacity to store data for 14 days, if the sensor is not reaching the smartphone or tablet [36].

At the baseline and 12-month follow-up visit the participants are instructed how to (1) position the sensor in the Band-Aid on the thigh, (2) download the SENS application to their smartphone or a tablet that is provided, and (3) connect the sensor to the app to upload the recorded data twice weekly. The participants receive Band-Aids and an instruction sheet including all relevant information.

### Secondary and exploratory outcome measures

#### Physical activity behavior

Beyond the proportion of participants that on average complete ≥ 8000 steps per day, physical activity is determined as the number of participants that on average complete ≥ 10,000 steps per day using the SENS motion® system. As recommended, we also use questionnaires in combination with the accelerometer data to gain more complete information regarding physical activity behavior [37]. We have chosen to use the Physical Activity Scale for the Elderly (PASE) questionnaire [38, 39] to provide descriptive data of the conducted physical activity.

*PASE* is one of the most commonly used self-reported instruments to assess physical activity in patients with OA [39], and it covers strength training and more stationary activities than can be measured by accelerometry [37]. PASE was developed to measure the level of physical activity (occupational, household, and leisure activities) over a 1-week period in persons aged 65 years and older [38]. The score range is 0–400 or more (lowest to highest physical activity level) [38].

Finally, we use the question: “How physically active are you now?” to grade the participants self-reported physical activity level (1 = almost entirely sedentary, 2 = light physical activity for 2–4 h per week, 3 = light physical activity >4 h per week or more vigorous physical activity for 2–4 h per week, 4 = more vigorous physical activity >4 h per week or regular heavy exercise or competitive sports several times per week) [40] and the question: “How many hours per week do you spend on physical activity that makes you breathless or sweaty?” to grade more vigorous physical activity (0=0 h, 1=½ h, 2=1 h, 3=2–3 h, 4=4–5 h, and 5=7+ h).

#### Core outcomes: physical function, pain, and patient global assessment

Core outcomes for clinical trials of OA are physical function and pain together with patient’s global assessment [23, 24]. Changes in physical function will be assessed using both “performance-based” and “patient-reported” outcome measures as recommended to obtain a complete picture of physical function [41, 42] (Fig. 1).

*“Performance-based” changes in physical function* is determined based on changes from baseline in the 6-min walk test (6MW), the 30-s chair stand test (30sCS), and a timed stair climb test (TSC) (Fig. 1). These test are included in a set of performance-based test recommended by the Osteoarthritis Research Society International that represents typical activities relevant to individuals following total joint arthroplasties [41]. Trained testers conduct the performance test and to the extent possible, each participant will be tested by the same tester at all time points. If a patient uses a walking aid in daily life (e.g., a rollator or crutches), she/he can use that during the functional performance test.

*The 6MW* covers the domain walking long distances and is also a proxy measure for endurance or aerobic capacity [43]. The 6MW measures the walking distance completed in 6 min on a 30-m flat, indoor course [44]. The participant is instructed to walk as far as possible in 6 min and is given standardized verbal encouragement at minute intervals [43, 44]. If required, a pause in a standing position is allowed. The 6MW has shown good reliability and acceptable agreement parameters in patients with hip OA [44, 45].

*The 30sCS* covers the domains: sitting and getting in/out of a seated position and is also a proxy measure for lower body muscle strength and power [43, 46]. This test measures the number of stands (to a fully extended position) from a straight-back chair without armrest (seat height 44.5 cm) completed in 30 s with hands crossed against the chest. The participant is instructed to complete as many chair stands as possible in 30 s [44, 46]. If the patient is unable to stand up from a chair without using the arms, a modified version where the patient can use the armrest is accepted. The 30sCS has shown good reliability and acceptable agreement parameters in patients with hip OA and after THA [44, 47].

*The TSC* covers the domains: mobility and climbing and is also a proxy measure for lower extremity muscle strength, power, and balance [43]. This test measures the time (best of two attempts) to ascend and descend a flight of 10 steps (step-height 16.3 cm, step depth 35.8 cm) without using the handrail [44]. The participant is instructed to ascend and descend the flight of stairs as fast as possible [44]. If the participant finds it unsafe or is unable to climb the stairs without using the handrail, a modified version with use of handrail is accepted. Stair climb tests have shown good reliability and acceptable agreement parameters in patients with hip OA and after THA [44, 47].

*“Patient-reported” changes in physical function* is determined based on changes from baseline in the function in daily living (ADL) subscale (17 questions) of the Hip disability and Osteoarthritis Outcome Score (HOOS) 2.0 [48, 49] (Fig. 1). The results have been shown to be useful for the evaluation of patient-relevant outcome after THA [49].

*The HOOS* questionnaire consists of five subscales: pain, other symptoms, function in daily living (ADL), function in sport and recreation (Sport/Rec), and hip-related quality of life during the last week [48, 49]. Standardized answer options are given (5 Likert boxes), and each question gets a score from 0 to 4 (4=worst score). A normalized score (100 indicating no problems and 0 indicating extreme problems) is calculated for each subscale [48, 49]. We have chosen the HOOS although it is only the second-best quality instrument to use in patients undergoing THA, because the highest-quality instrument the Western Ontario and McMaster Universities Osteoarthritis Index (WOMAC) requires license to use [50].

*Changes in pain* is determined based on changes from baseline in the subscale pain of HOOS (10 questions) [48] (Fig. 1).

*Changes in patient’s global assessment* is assessed as transition ratings of global perceived effect where the participants are asked to compare their current global wellbeing with the pre-surgery state (Fig. 1). We have designed a transition questionnaire on a 200-mm visual analog scale with anchors being: −100 = “Much worse”; 0 = “No changes”; 100 = “Much better”.

#### Health-related quality of life

Changes from baseline in health-related quality of life are assessed with a generic instrument the EuroQol 5-Dimension Questionnaire (EQ-5D-3L) and a disease-specific instrument the HOOS subscale hip-related quality of life (Fig. 1).

*The EQ-5D-3L* [51] consists of two parts: a 5-item descriptive system (EQ-5D) and a thermometer-like visual analog scale (EQ-VAS) that records the patients self-rated health on a 20-cm vertical VAS, where the end points are labelled “worst imaginable health state” at 0 and “best imaginable health state” at 100. The descriptive system comprises 5 dimensions (mobility, self-care, usual activities, pain/discomfort, and anxiety/depression), and each dimension has 3 levels (L): no problems, some problems, and extreme problems [52]. Each question/dimension is assigned a score from 1 to 3 which can be summarized to produce 243 health states, also known as the health profile [52]. The EQ-5D-3L has been validated in Danish [53] and has been widely used and tested in various population groups including patients having total joint replacement [54], and Danish population norms are available [55]. A newer EQ-5D-5L survey has shown stronger validity evidence and may allow more sensitive measurements of change in patients undergoing THA [54, 56], but Danish population norms are not available.

*The subscale hip-related quality of life of HOOS* consists of 4 questions [48].

#### Exploratory outcomes: self-efficacy and outcome expectations

The combination of self-efficacy (the individuals’ beliefs in their capability to perform a specific behavior) and outcome expectations (a person’s estimate that a given behavior will lead to certain outcomes) are believed to predict behavior [57]. We determine changes from baseline (Fig. 1) using the exercise self-efficacy scale and “task-specific self-efficacy” and the Outcome Expectancy for Exercise scale-2 (OEE-2).

*The exercise self-efficacy scale* [58] is a five-item self-efficacy measure designed to measure confidence in one’s ability to persist with exercise, when challenged by known barriers to exercise [58]. Items represent the following areas: when - I am tired, I am in bad mood, I feel I don’t have the time, I am on vacation, and when it is raining or snowing [58]. A five-point scale is used to rate each item, with 1 indicating “not at all confident” and 5 “very confident”.

*“Task-specific self-efficacy”* is measured in conjunction with the TSC test [59]. After a practice trial, the participants are presented with a 10-step confidence ladder ranging from 0 (completely uncertain) to 10 (completely certain) and asked to rate the level of certainty that they can complete the stair-climbing task 2, 4, 6, 8, and 10 times without stopping (total score 0–100 (worst to best)) [59].

*The 13-item OEE-2* [60] includes both positive (nine items serving as a positive outcome expectancy for exercise subscale (POEE)) and negative (four items serving as a negative outcome expectancy for exercise subscale (NOEE)) expectations associated with exercise. To complete the OEE-2 scale, the patient is asked to listen to each statement about outcomes of exercise and to rate each of the thirteen items out of 1=strongly disagree to 5=strongly agree [60]. The POEE and NOEE subscales are scored by calculating the average score on each scale and the items in the NOEE are reversed before the scores are summed [60]. A higher score indicates more positive outcome expectations regarding exercise [60].

#### The patients’ perspective

To capture the patients’ experience with the PANORAMA intervention, we will conduct semi-structured focus group interviews with participants who have completed the intervention. The interviews will focus on themes: capability, opportunity, and motivation [22] and will be analyzed using a deductive-inductive approach.

### Additional information

Before surgery, the following data are collected from the patients using an interviewer-administered questionnaire developed for the trial: age, sex, height, body weight, marital status/living together/living alone, any joint replacement in the lower extremities, joint pain comorbidity (lasting ≥ 3 months), comorbidity/chronic diseases, current physical activity, and exercise behavior. The latter includes a questionnaire with the question “How physically active are you now?” [40] and the same question when < 20 years, 20–30 years, 30–40 years, and so forth to give an impression of the patient’s life-time physical activity behavior. In addition to the OEE-2, the patients fill out the following questionnaires: the HOOS, the EQ-5D-3L and EQ-VAS, the PASE, and the exercise self-efficacy scale (Fig. 1).

At 3 (baseline), 6, and 12 months after THA, the patients are asked about occurrence or absence of hip, knee, or back pain using a questionnaire developed for the trial. In case of pain, the patients are asked to rate how limited (not at all, mildly, moderately, severely, extremely) they are because of their hip, knee, or back pain. We have selected hip, knee, and back pain because it has been demonstrated that patients with hip OA often suffer from knee and back pain [61] and that pain in other joints especially back pain has shown to negatively impact outcomes after THA [11, 12].

The participants height (only 3 months after surgery) and weight (light clothing, shoes removed) are measured by the tester at all postoperative time points immediately before the functional performance test.

### Sample size

The sample size was determined to detect a difference where at 6 months 30% in the control group will have reached an average daily number of steps of 8000 spontaneously and 50% of participants in the intervention group will have reached a daily average of 8000 steps. With an allocation ratio of 1:1 and a two-sided significance level of 0.05, a chi-squared test needs a total sample size of 200 to achieve an approximate power of 0.83 for this effect size; this corresponds to a number needed to treat of 5 patients.

### Randomization

Randomization is performed according to the order in which the participants have completed baseline test (Figs. 1 and 2). The participants are allocated in a 1:1 ratio (immediately after baseline assessment) to receive either the PANORAMA intervention or control by using envelope-based randomization. To control for potential imbalance in the randomization, stratification for self-reported pre-surgical physical activity level (high pre-surgical physical activity level Yes/No), age (≥75 years/<75 years), and total joint replacement in the lower extremities within the last 12 months (yes/no) is employed.

We have chosen to stratify for pre-surgical physical activity level because a great variability in physical activity habits in patients with severe OA has been demonstrated [62]. The baseline (pre-surgery) physical activity level may affect the rate of change in physical activity [63]. Information about self-reported pre-surgical physical activity level will be collected with the question “How physically active are you now?” [40] and level 3–4 is determined as high pre-surgical physical activity level (yes) while 1–2 is not (no).

Age (≥75 years/<75 years) has been chosen because younger adults are generally more physically active than older adults. Data from the Danish Health Authority’s publication “The Danes health. The national health profile 2017” [64] has shown that the proportion of Danes who do not meet WHO’s minimum recommendation for physical activity (do at least 150 min of moderate-intensity aerobic physical activity throughout the week) is highest among older adults aged ≥ 75 years.

Finally, we have chosen to stratify for total joint replacement in the lower extremities within the last 12 months (yes/no) because participants who have undergone total joint replacement in the lower extremities within that period are expected to be more deconditioned and have lower physical activity level than those who have not undergone surgery within the last 12 months.

### Allocation—concealment mechanism

Prior to the study computer-generated allocation, sequences (one for each case of stratification, 2×2×2=8 lists) were provided by an external statistician not otherwise involved in the study. The allocation list is stored on a password-protected computer drive of an independent researcher who is not involved in the study. Each individual allocation in the sequence has been cut out and concealed in opaque envelopes and placed in eight folders of each 120 envelopes containing the written message “intervention” or “control” by the same researcher. The order of the content of the envelopes corresponds to the randomization list. The envelopes are numbered consecutively from 1 to 120 and placed in the folder accordingly with number 1 in front and number 120 in the back. The same researcher has checked that the envelopes are sealed and opaque and the folders are kept in a locked cabinet in department of physical and occupational therapy. When a participant has completed the baseline assessment, a member of the staff not involved in the recruitment, enrollment, or assessment of the participants picks an envelope from the folder corresponding to the stratification starting with the envelope in front. The participants themselves open the envelope.

### Blinding

All assessors/testers are blinded to group allocation and previous test results. Due to the nature of the study, the patients and the physiotherapists who deliver the PANORAMA intervention cannot be blinded, but the patients are requested not to reveal their group allocation to the blinded outcome assessors. The biostatistician (VS) who performs the data analyses and validates the results will be blinded to group allocation. Since the principal investigator is not blinded to group allocation, emergency unblinding will not be needed.

### Data collection, management, and analysis

#### Data collection and management

Data management will comply with the rules of the Danish Data Protection Agency (approval reference number: P-2020-75). Standardized protocols for the data collection process and procedure to be conducted have been developed and all involved study personnel have been trained to ensure that procedures and recording of data and results are standardized. All paper case report forms and questionnaires are checked for errors and missing data before being archived in a study database and all paper-based versions are locked in a filing cabinet. Upon completion of data entry, the study database will be checked against the paper-based assessments to ensure acceptable accuracy and completeness. When all participants have reached the primary respectively the secondary end point, the physical activity data will be exported from the SENS’ database through SENS innovation service and entered into the study database.

#### Data retention plan

To improve participant retention and prevent missing data the following procedures are established: (1) all appointments 3, 6, and 12 months after surgery will be arranged via phone calls supplemented with written information 2 to 4 weeks in advance. In addition, the participants receive a text message reminder 1–2 days before their appointment; (2) study staff will check if participants regularly upload data from their physical activity sensor via the SENS motion® system. If data are not uploaded in 7–8 days, the participants are contacted by phone to ensure that they have no technical issues, and to remind them to upload data; (3) after the participants have completed the questionnaires, the tester will ensure that all the questions are answered; (4) after completion of the assessment at the 12-month follow-up, all participants will receive their results for the functional performance test from all the assessments.

#### Data monitoring committee

It is not expected that study participation will be associated with risks or complications thus no data monitoring committee has been established.

#### Statistical analysis

A description of the study population and an assessment of the randomization success is provided by tests for the difference between the randomization groups of baseline values for the outcomes and the selected covariates. The primary effectiveness analyses performed are intention-to-treat (ITT) assessments of the between-group difference in the outcomes at 6 months after surgery, beyond a difference already present at baseline 3 months after surgery; similar analyses assess the between-group differences at 12 months. These analyses are performed in longitudinal logistic regression models for binary outcomes—such as the primary outcome—and longitudinal linear regression models for continuously valued outcomes. Potential differential dropout between the groups is adjusted for with inverse probability weighting, the weights estimated by multivariable logistic regression models. Both the repeated measurements and the weighting are adjusted for with the method of generalized estimating equations (GEE).

### Risks and side effects

According to the current good clinical practice standard, passive surveillance of harms will be assessed, i.e., the recorded adverse events are those that the study participants spontaneously report on their own initiative. There is a small risk of allergic reaction to the Band-Aids holding the SENS accelerometer. If this should happen, the participant will receive specific skin care. Patients are covered by the “patient-compensation agreement” if anything unexpected should happen during the intervention.

### Protocol amendments

The principal investigator will inform the Research Ethics Committee and the Data Protection Agency if significant changes in the protocol occur and make an update at ClinicalTrials.gov.

### Dissemination

All results, regardless whether positive, negative, or inconclusive, will be published in scientific peer-reviewed journals, with authorship following the International Committee of Medical Journal Editors (ICMJE) guidelines for publication.

## Discussion

The PANORAMA trial investigates an important but sparsely researched clinical question concerning effective management strategies of promotion and support for physical activity after THA. The trial is based on the assumption that a behavior change intervention is required and on research results suggesting that using a multicomponent intervention results in better physical activity adherence [65]. None of the previous studies investigating the effects of adding specific interventions to increase physical activity after THA or TKA [17–21] have addressed patient-reported barriers to physical activity. According to psychological health behavior change theories, it is important to consider barriers and facilitators in order to describe and gain a comprehensive understanding of an individual’s physical activity behavior [66]. Beyond uncertainty, patients have highlighted experience of pain in other joints or other limitations such as age and comorbidities as barriers to physical activity after total joint replacement [13, 14]. Patient-reported key facilitators to increase physical activity uptake include offers of individualized physical activity linked to social integration and activities that are perceived as pleasurable rather than merely benefiting health [13]. Furthermore, encouragement or the lack of encouragement to exercise by a physician has been shown to be important for exercise or physical activity behavior in patients with OA [67–69]. Finally, central determinants of physical activity behavior in general include a number of personal factors: individual attitudes, skills, emotions, beliefs, and knowledge [15, 66, 68]. Physical inactivity is more often depending on personal factors, such as emotions, beliefs, self-regulation skills, self-efficacy, and motivation than on the functional impairments imposed by a medically defined disease [66].

Feedback from physical activity trackers or pedometers in combination with, e.g., goal setting have shown promising results for increasing physical activity after total joint arthroplasty [17, 18, 21] and strong evidence for effectiveness to increase physical activity in patients with musculoskeletal diseases [70]. However, there is still a gab in our understanding of the relationship between the nature of goal setting and participant success [63]. Even though it is tempting to prescribe a specific total or gradual increase number of steps per day an individualized program is more personally relevant, easily adjustable, and likely to be more feasible for typically sedentary individuals [63].

The overall aim of the PANORAMA intervention is to empower the patient to autonomously achieve favorable behavior changes. For optimal effect, the study applies a sound theoretical basis targeting the appropriate behavioral change determinants [66], and patients have been involved in the development. Based on our preliminary interviews with the patients, we believe that 3 months after THA could be a window of opportunity to change patients’ attitudes and regular physical activity behaviors. At this time point, most of the patients have completed outpatient physiotherapy, and according to them, completed outpatient physiotherapy could represent a pitfall for physical inactivity.

Physical activity is a complex behavior and challenging to measure [37]. Currently, there is no consensus on how physical activity should be measured and reported in patients undergoing total joint replacement or other orthopedic patient populations [71]. Even in accelerometry studies investigating changes in physical activity after THA or TKA, there is no consensus regarding outcome measures; whether it should be step counts, percentage of daily movement-related activity, hours per day spent lying, sitting, standing, and walking, daily minutes with light, moderate and vigorous physical activity, and so forth [4, 5].

Patients with knee or hip OA show a large variation in physical activity habits, regardless whether they have undergone joint replacement surgery or not [4, 5, 62]. A contributing factor to this variability could be that the populations studied also differed by age, country of residence, type of joint replacement, time after surgery, and body mass index [5]. However, even after considering these between-study differences, the magnitude of variance in reported physical activity, e.g., step counts, is still difficult to explain [5].

We have chosen step counts as the primary outcome. Various step-based versions or translations of physical activity guidelines have emerged, and 8000 steps per day has been calculated to represent a minimal or floor value of recommended physical activity [72]. In addition, walking more than 6000 steps per day have been suggested as a level of walking to protect against developing functional limitations in persons with knee OA [73].

Two studies [21, 74] have reported steps per day prior to and after THA in persons over 50 years. One observational study reported that the patients (mean age 61 years) completed 4632, 5657, and 6163 steps per day respectively pre-surgery and 6 and 12 months post-surgery while healthy peers completed 7228 steps [74]. The other study [21] investigated the effect of supplementation with a pedometer-driven intervention the first 6 weeks after THA or TKA. Pre-surgery the intervention group (mean age 67 years) completed on average 7069 steps per day and 6 months post-surgery 8326 per day and at that time point 70% of the patients completed ≥ 7000 steps per day [21].

The aim of the current study is to investigate the effect of a multimodal physical activity promotion/education intervention under realistic clinical conditions. Accordingly, we have used a more pragmatic approach and study design [75] (Fig. 3). Given the more pragmatic design, it can be argued that the study environment is not completely normal. However, we believe that the hospital-based orthopedic department is ideal for recruiting patients and delivering the intervention. This is because all patients undergoing a THA (*n* = 452 at BFH in 2017) are examined by a physician in the outpatient department preoperatively, and this provides an opportunity to utilize already established procedures, including access to preoperative data. In addition, the close interdisciplinary collaboration between surgeons and physiotherapists is an advantage, because both groups give advise regarding physical activity after surgery.

By applying a pragmatic approach, we have used broad participant eligibility criteria and not just restricted study participants to those likely to be highly responsive to the intervention or to have favorable outcomes. Thus, we include patients with comorbidities if they are home-dwelling, independent, and self-reliant even though it has been demonstrated that comorbidities could be a barrier for physical activity uptake [13] and have a negative impact on outcome after THA [11, 12]. However, the broader eligibility criteria are likely to increase the generalizability and applicability of the study results.

If this RCT shows that the PANORAMA intervention can increase physical activity after THA and further translate into additional improvement in physical function, it could be a relatively inexpensive method for potentially improving general health among the patients and decrease healthcare costs. Protocols for the counselling and the patient educational material will be available after completion of the study. Thus, it should be possible to implement the PANORAMA intervention in other clinical settings both in Denmark and abroad with few adjustments.

## Trial status

**Table:** 

Protocol version (date)	1 (31 July 2019)
Date of first enrolment	31 August 2020
Completed recruitment	30 December 2022
Recruitment status	Recruiting

## Data Availability

TB will have access to the final trial dataset. The datasets generated during the trial will not be publicly available due to regulations set out by the Danish Data Protection Agency.

## Abbreviations

OA: Osteoarthritis
THA: Total hip arthroplasty
TKA: Total knee arthroplasty
RCT: Randomized controlled trial
BFH: Bispebjerg and Frederiksberg Hospital
TB: Theresa Bieler
MR: Mie Rinaldo
BCT: Behavior change technique
AK: Anne-Mette Krifa
PASE: The Physical Activity Scale for the Elderly
6MW: The 6-min walk test
30sCS: The 30-s chair stand test
TSC: Timed stair climb test
HOOS: The Hip disability and Osteoarthritis Outcome Score
WOMAC: The Western Ontario and McMaster Universities Osteoarthritis Index
EQ-5D: The EuroQol 5-Dimension Questionnaire
VAS: Visual analog scale
L: Level
OEE: Outcome expectancy for exercise scale
NOEE: Negative outcome expectations for exercise subscale
POEE: Positive outcome expectations for exercise subscale
VS: Volkert Siersma
CRF: Case report form
